# Reference Point Heterogeneity

**DOI:** 10.3389/fpsyg.2016.01347

**Published:** 2016-09-12

**Authors:** Ayse Terzi, Kees Koedijk, Charles N. Noussair, Rachel Pownall

**Affiliations:** ^1^Department of Finance, Tilburg UniversityTilburg, Netherlands; ^2^Department of Finance, Tilburg UniversityTilburg, Netherlands; ^3^Department of Economics and Economic Science Laboratory, University of ArizonaTucson, AZ, USA; ^4^Department of Finance, Tilburg UniversityTilburg, Netherlands

**Keywords:** reference point, experiment, decision making, risk

## Abstract

It is well-established that, when confronted with a decision to be taken under risk, individuals use reference payoff levels as important inputs. The purpose of this paper is to study which reference points characterize decisions in a setting in which there are several plausible reference levels of payoff. We report an experiment, in which we investigate which of four potential reference points: (1) a population average payoff level, (2) the announced expected payoff of peers in a similar decision situation, (3) a historical average level of earnings that others have received in the same task, and (4) an announced anticipated individual payoff level, best describes decisions in a decontextualized risky decision making task. We find heterogeneity among individuals in the reference points they employ. The population average payoff level is the modal reference point, followed by experimenter's stated expectation of a participant's individual earnings, followed in turn by the average earnings of other participants in previous sessions of the same experiment. A sizeable share of individuals show multiple reference points simultaneously. The reference point that best fits the choices of the individual is not affected by a shock to her income.

## 1. Introduction

Economic decision making under risk involves the consideration of the probabilities of various outcomes, as well as the perceived utilities of these outcomes. However, empirical work has suggested that when judging and evaluating a risky lottery, reference payoff levels are also critical. A payoff appears to be evaluated based on how it compares to a reference level, with a reference point serving to separate desirable from undesirable outcomes, according to some criterion. Thus, understanding how payoff levels come to be viewed as reference points is a key step in uncovering the cognitive process that generates decisions taken under risk.

Indeed, reference dependence, an asymmetry in the treatment of payoffs above vs. below a benchmark payoff level, has been a robust finding in both economics and psychology, since it was first proposed and documented by Kahneman and Tversky (1979). Reference dependence is a cornerstone of prospect theory, the most influential behavioral model of decision making under risk. Reference points have been shown to characterize decision making in laboratory research, surveys, and in field data from numerous domains. These domains include household saving, labor market participation, consumer behavior, education, and investment decisions (see e.g., Hardie et al., 1993; Camerer, 1997, 2004; Starmer, 2000; Grinblatt and Han, 2005). Experimental studies have documented the effect of reference point formation on the provision of effort (Abeler et al., 2011), the pricing of securities (Tversky and Kahneman, 1992), and the exchange and valuation of consumer products (Ericson and Fuster, 2011).

However, while there is general agreement that reference points are important, little is known about which payoff levels will come to serve as reference points. Typically, in empirical work, the reference points of the decision maker are taken as evident given the decision context. This is reasonable in some settings, though less plausible in others. There are no widely-accepted, general accounts of how a particular payoff level emerges as a reference point.

Furthermore, it is not clear that in a particular given decision context, only one unique reference point is relevant. Kahneman (1992) raises the possibility of multiplicity of reference points and characterizes this as an important topic for future study. Sullivan and Kida (1995) demonstrate that corporate managers form multiple reference points, specifically the historical profit level, as well as profit and revenue targets. In an experimental study, Baucells et al. (2011) show that the reference trading price of a financial asset is a combination of multiple potential reference prices.

One class of prominent theories of reference point formation is based on the expectations of the decision maker herself (Bell, 1985; Loomes and Sugden, 1986; Kőszegi and Rabin, 2006, 2007; Heidhues and Kőszegi, 2008). Expectations-based reference points have been used to explain insurance choices (Barseghyan et al., 2011), and labor supply decisions (Farber, 2005, 2008; Crawford and Meng, 2011). However, the payoffs that peers receive are also relevant. Experimental work has largely supported the models of inequity aversion proposed by Fehr and Schmidt (1999) and Bolton and Ockenfels (2000), which assume that the average payoff of peers serves as a reference point. Furthermore, expectations can be formed through a history of social interaction, e.g., contracts, experiences, past trends, or the recommendations of others (Davies and Kandel, 1981; Abel, 1990; Gali, 1994; Carmeli and Schaubroeck, 2007; Vendrik and Woltjer, 2007; Post et al., 2008; Linde and Sonnemans, 2012). Kőszegi and Rabin (2006) point out that there are multiple candidates that can serve as expectation-based reference points. They emphasize that candidate reference points might also coincide. For example, the expectations of an individual about her own and her peers' payoffs may be the same in some instances. The reference point in effect is obviously consequential. For example, Kőszegi and Rabin (2007), argue that the implications of reference dependence differ depending on the specification of the reference point.

Thus, there are several candidate expectation-based reference levels that appear to be prominent. The purpose of the paper is to study which reference points characterize decisions in a setting in which there are several plausible reference levels of payoff. The question we consider here is individuals differ from each other in their propensity to use different reference points, when they make decisions in the same setting. We study which, if any, of four candidate reference points is most likely to emerge in a decontextualized setting. If the reference points that emerge vary greatly by individual, it can only be due to differences arising from the individuals themselves, rather than the task or the setting.

To investigate this, we conduct an experiment which allows a participant to use any or all of four competing reference points in a risky decision making task. The first is the payoff level for the individual anticipated by the experimenter (who may be interpreted as an authority figure or an employer). We abbreviate this reference point as IE, or Individual Expectation. This level, indicated on each subject's instructions, is a natural candidate for a reference point, since it directly ascribes a benchmark for the individual to attain. The second potential reference point is the anticipated average payoffs of peers in the same decision situation (PE, Peer Expectation). This is also indicated in writing on an individual's instructions, with equal prominence as IE. Note that expectations, as used here, do not refer to an individual's own beliefs or aspirations, or to a mathematical expectation of her payoff. The third is the historical average payoff of others in the same position in past sessions (HA, Historical Average), also indicated in the instructions, and the fourth is the average performance of a relatively large population (PA, Population Average), which is known to subjects at the time of recruitment to the session. PE, HA, and PA all represent payoffs of other individuals in the same or similar experiments, but vary in the social distance between the parties they apply to and the individual herself. Because there is no compelling rationale for believing that one reference point would dominate the others, we refrain from advancing hypotheses in advance about which reference points would be most consistent with the data.

In our experimental design, we present three of the reference points simultaneously, in order to conduct a horse race between the alternatives. In some session we presented PA, IE, and HA, while in others session the payoff levels displayed were PA, IE, and PE. We elicit the certainty equivalents of a large number of lotteries and obtain estimates of individual reference points. The design permits the detection of individuals who use none or one unique reference point, as well as those who employ multiple reference points concurrently. By using one fixed probability for gains and losses of 0.5 throughout the experiment, we attenuate the impact of probability weighting on our results.

It is also important to understand whether reference points change in response to shocks to wealth levels. Some studies have considered this topic. Arkes et al. (2008) show that subjects are more likely to adapt their reference points to gains in their wealth than to losses. Chen and Rao (2002) stress the importance of the order of presentation of two equally-sized gains and losses. They suggest that the first payoff that is presented leads to a more significant adaptation of the reference point than the second. In a financial market setting, Baucells et al. (2011) show that reference prices for a financial asset are a function of the first and the last trading price. Masatlioglu and Ok (2005) model the theory of choice in a static setting where the initial endowment or status quo plays a key role. They show that an agent with reference-dependent preferences prefers to stay at his status quo as long as another option does not dominate it in all dimensions. Post et al. (2008) find evidence of path dependence in reference levels in choices under risk. One of the treatments in our experiment is complementary to this strand of research, and allows us to study the adjustment of the reference point after a shock to one's income level.

Our results show that if all individuals are classified by the one reference point that they adhere to most closely, the population average (PA) is employed most frequently followed by the individual expectation (IE), and then by the historical average (HA). The social comparison group which is the most distant though also the largest, the population of experimental subjects, appears to be the most relevant. Multiple reference points are observed for a sizable share of individuals, while some others show no evidence of having any reference point. Many individuals use a heuristic, in which they value a lottery at a fixed percentage of its expected value. Finally, we find evidence that reference points do not change after a shock to income has occurred. Overall, these results reveal that there is individual-level heterogeneity in the use of reference points within a fixed decontextualized setting. Thus, reference point choice is driven in part by personal inclination.

The remainder of this paper is organized as follows. Section 2 describes the experimental design. In Section 3 we discuss the results, and Section 4 concludes the paper.

## 2. Materials and methods

### 2.1. Conduct of sessions and procedures

A total of 44 sessions were conducted at the Centerlab at Tilburg University in The Netherlands, between November 2013 and June 2014. Subjects were all Bachelor's and Master's students in Economics and Business Administration, and therefore were relatively homogeneous in their training. A total of 163 subjects participated. Fifty-five percent were male. The average age of member of the subject pool is 22. The experiment was executed with the z-Tree computer program (Fischbacher, 2007). There was a varying number of participants per session and each subject acted independently of others in this individual decision making experiment. Each session lasted 45 min, including the time during which the experimenter read the instructions. The payoffs in the experiment were expressed in terms of an experimental currency, which was converted to a Euro payment to subjects at the end of the sessions. The average earnings per subject were 16 Euros (1 Euro = $1.30 approximately at the time the experiment was conducted).

A session consists of 60 periods. In each period *t*, subjects are presented with a binary prospect (1/2, *y*_*t*_), which results in outcome *y*_*t*_ with probability 0.5 and in outcome 0 with probability 0.5. This prospect is paired with eight different certain payment levels, *x*_*jt*_, *j* = 1, …, 8 in a price list format, during each of the 60 periods. In each period, each subject must make eight choices. Each choice in period *t* is between (1/2, *y*_*t*_) and *x*_*jt*_. The eight choices are displayed on the subject's computer screen simultaneously. The magnitude of *x*_*jt*_ ranges in value from 40 to 180% of *y*_*t*_/2, the expected value of the prospect. The certain payments appear in ascending order of magnitude in the price list on the computer screen.

The sixty periods are divided into three 20-period segments. The certain payments *x*_*jt*_, as well as the amount that the lottery can pay out *y*_*t*_, increases in constant increments from one period to the next within each segment. The lowest certain amount *x*_*jt*_ chosen by the subject over (0.5, *y*_*t*_) in period *t*, serves as our measure of the certainty equivalent for the prospect (0.5, *y*_*t*_) for that subject. The expected value of the prospects and the potential certainty equivalents span the four potential reference points. Thus, the expected values of (0.5, *y*_*t*_), as well as the value of *x*_*jt*_, are in some instances in the domain of gains and at other times in the domain of losses relative to each of the four reference points we consider.

At the beginning of a session, the experimenter read the instructions for the experiment aloud. The instructions included key statements about earnings, which were intended to introduce the candidate reference points.

Subjects registered through an online system and at that time were informed of the average earnings in Euros for experiments of similar length conducted at the laboratory, 12 Euros. This is the overall average payoff of subjects participating in an experiment at Centerlab, and we interpret this level as the PA reference point.

At the start of the experiment, each subject was given information about his/her initial cash balance, which was hers to keep. This information remained on her computer screen for the duration of the session. The initial balance was always less than the PA reference level. Therefore, to reach the PA level, the subject had to earn the difference between this level and the initial balance.

The level of the IE reference point was indicated in bold font on the instructions that subjects received at the beginning of the session. It was also displayed on participants' computer screens for the entire session. It was emphasized that this individual expectation was not based on any specific knowledge about the realized final outcome, but only about what could be expected beforehand based on the way the experiment was designed.

In sessions 2–24, the historical average of earnings of participants from previous sessions of the experiment (the HA reference point) was also emphasized in the instructions and indicated on the computer screens. In sessions 25–44, the PE reference point was presented similarly.

We varied the magnitudes of the four reference points in different sessions. The values of each of the four candidate reference points are shown in Table 1. The first column of Table 1 indicates the session, and each row groups together sessions conducted under identical parameters. The next three columns contain the monetary values, in terms of experimental currency, of each of the reference points. All four reference points are net of the initial endowment, which differs by individual. The PE and IE were adjusted to reflect the different parameters in effect in different sessions, and the HA differed because earnings of individuals in previous sessions varied. Each reference point was always a at a unique value for an individual subject, and the intervals in the table indicate the range of differing unique reference points among subjects in the session indicated. The ranges within each session are indicated in columns 2 and 3. Columns 5 and 6 give the exchange rate between experimental currency and Euros in effect, and whether there was an income shock after period 40. The payoffs were denominated in terms of an experimental currency that was convertible to Euro at the end of the session, at a conversion rate indicated in the second-to-last column of Table 1.

**Table 1:** **Parameters used in the experiment**.

**Session**	**Initial balance**	**Expectation of own earnings (IE)**	**Historical Average (HA)**	**Expectation of Peers (PE)**	**Population Average (PA)**	**Exchange rate**	**Treatment**
1^*^	3500–6500	5500–8500	–	–	9100–12,100	1300	Baseline
2–3	4500–7000	7000–9500	15,600	–	8600–11,100	1300	Baseline
4–5	4500–7000	7000–9500	13,500	–	8600–11,100	1300	Baseline
6–7	4500–7000	7000–9500	12,700	–	8600–11,100	1300	Baseline
8–10	8500–10,500	15,500–17,000	13,100	–	7500–9500	1500	Baseline
13–24	35,000–45,000	45,000–60,000	28,500	–	33,000–43,000	6500	Baseline
25–32	50,000–60,000	70,000–85,000	–	100,000	42,000–52,000	8500	Shift
33–44	50,000–60,000	70,000–85,000	–	100,000	48,000–58,000	9000	Shift

* *Session 1 is excluded from the analyses due to the absence of a historical average. IE is the earnings level that the experimenter indicates to individual that is expected of her. PE is the earnings level that the experimenter indicates to an individual that he/she expects others participating in the same session to earn. HA is the average earnings of individuals in all prior sessions. PA is 12 Euros, the average earnings in all experimental studies conducted at the laboratory, minus the initial endowment. All reference points are similarly expressed net of the initial endowment and income shock. Within a session different individuals had different initial balances, IE and PA reference points. Thus, the indicated values are ranges. However, each individual himself had a unique initial balance, IE and PA level*.

At the end of the session, the computer randomly chose one period *t* and one of the decisions within that period to count as each subject's earnings. Depending on the choice of the subject, the subject either played the lottery and received one of the outcomes of the prospect, 0 or *y*_*t*_, or obtained the certain amount *x*_*jt*_^1^.

### 2.2. Treatments

There were two treatments in the experiment, called Baseline and Shift. The last subsection described the Baseline treatment. In the sessions of the Shift treatment, we induced an exogenous shock to income after the 40th period by paying a bonus that was unanticipated by subjects. The bonus for each individual was equal to 50% of the initial endowment. It was emphasized that the shock was independent of the earlier choices participants made. The shock was described to participants by the following announcement made by the experimenter before period 1. “If during the course of the experiment any new information will be shown to you on the screen, please note that this is not due to the decisions you have previously made in the experiment. The computer does not do anything with your decisions until the experiment finishes.”

## 3. Results

This section is organized in the following manner. We first informally describe the data from two typical subjects. Section 3.1 describes and documents the widespread use of a rule, called the Proportional Discounting Heuristic, employed by 38% of our participants. Section 3.2 contains our analysis of the prevalence of the four different reference points.

**Figure 1:**
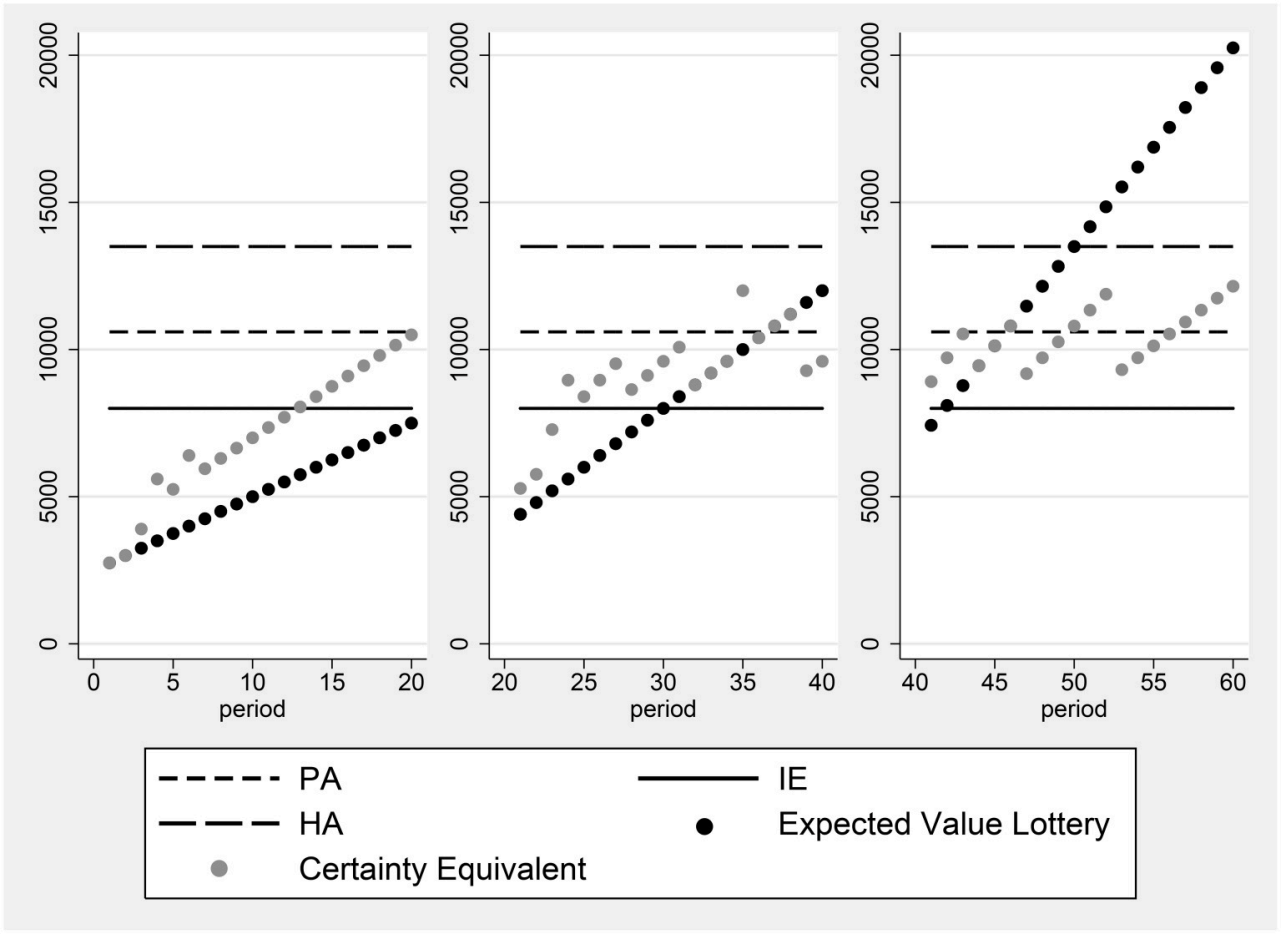
**Certainty equivalents of subject 16, who participated in session 5**.

**Figure 2:**
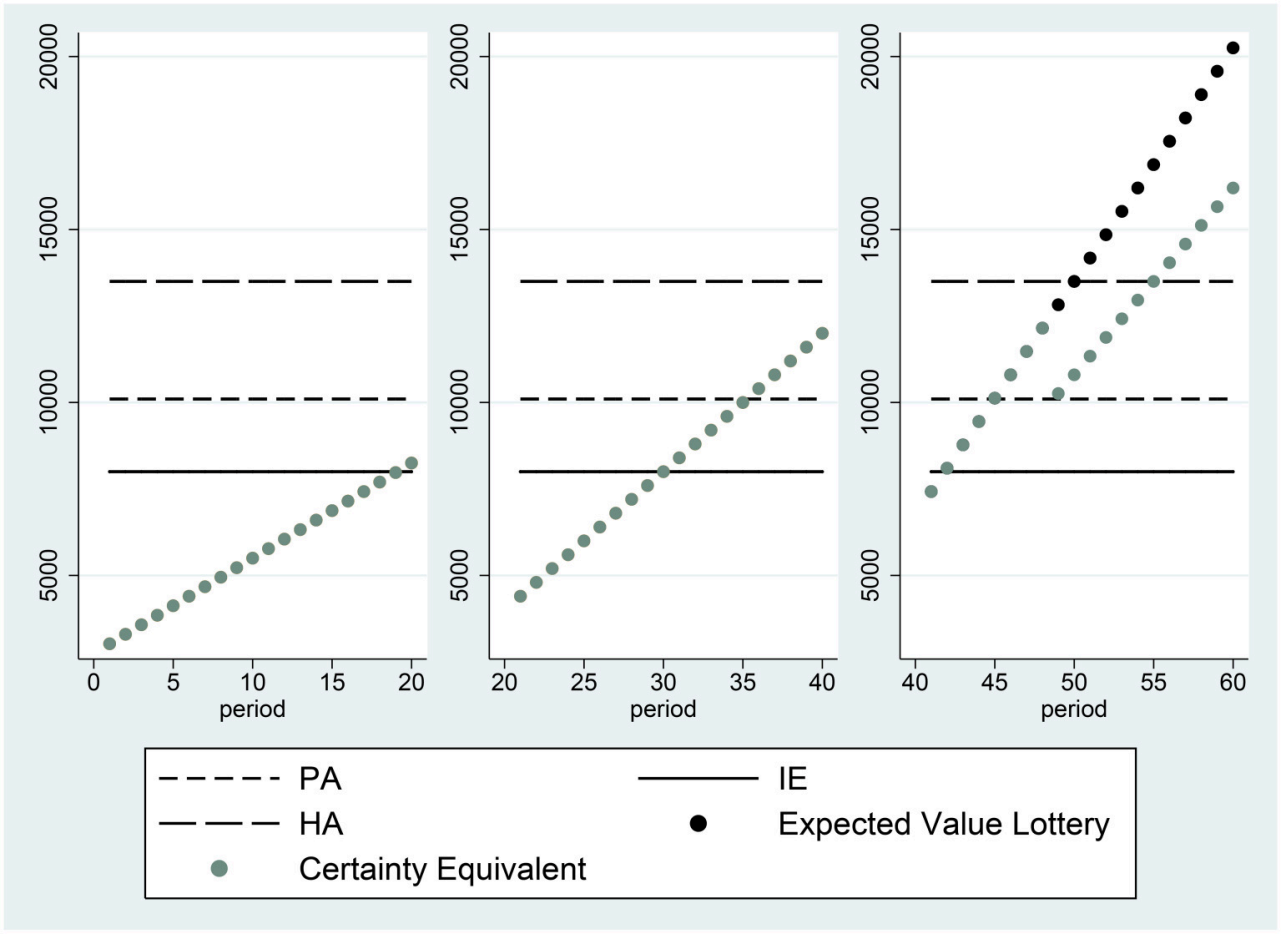
**Certainty equivalents of subject 13, who participated in session 3**.

Figures 1, 2 illustrate two of the typical decision profiles in our data. The horizontal axis gives the period number, while the vertical axis shows monetary amounts expressed in terms of experimental currency. The points displayed in black are the expected values of the prospects presented in the period indicated. The certainty equivalents elicited from the subject in the period are given by the gray points. The leftmost panel shows the expected values of the prospects and the certainty equivalents elicited in the first 20 periods. The expected values of these prospects include values both above and below a candidate reference level. The figure shows that the certainty equivalents of subject 16, who is depicted in the figure, are greater than the expected value of the prospects, whenever the expected value lies in the domain of losses relative to the PA reference point. Thus, the subject exhibits risk seeking behavior in this domain. When the expected value of the lottery lies above the PA, the observed certainty equivalents are less than the expected value of the prospects, which is consistent with risk averse preferences. Thus, we observe here that the subject changes her attitude toward risk at the PA payoff level^2^.

Another example, for subject 13, is presented in Figure 2. The certainty equivalents of this subject are all equal to the expected value of the prospect, whenever the expected value of the prospect is less than the Historical Average. This indicates that the individual is risk neutral in the domain of losses, relative to the HA reference point. When the expected value of the prospect is greater than HA, the individual becomes risk averse.

### 3.1. The proportional discounting heuristic

**Figure 3:**
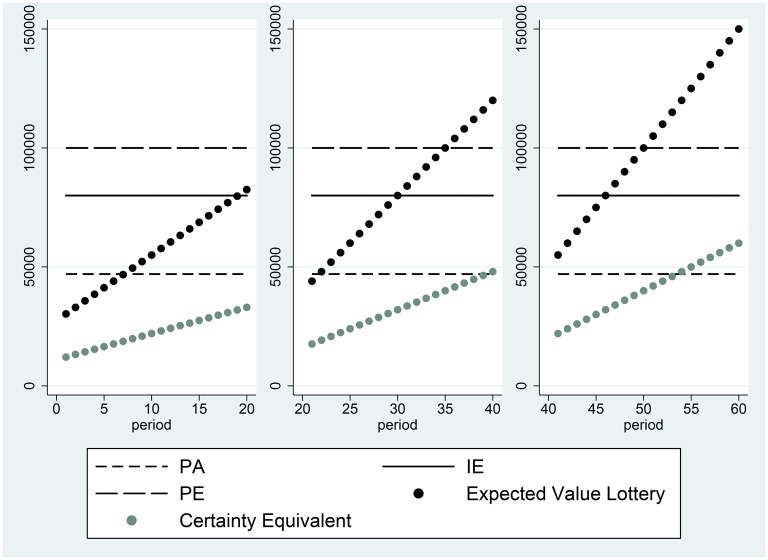
**Certainty equivalents of subject 100, who participated in session 32 and did not employ a reference point**.

A very common decision rule, employed by 38% of individuals, is the Proportional Discounting Heuristic. This rule involves setting a certainty equivalent equal to a constant fraction of the expected value of the lottery (or alternatively to a constant fraction of the maximum possible outcome of the lottery), as is depicted in Figure 3. The agent depicted in this figure has no reference point in the range spanned by the possible certain payments offered in the experiment (although we cannot rule out the possibility that the agent has a reference point at 0, for example). The certainty equivalent of individuals who proportionally discount is given by:

(1) $$\begin{array}{r} Certainty\;equivalent=\alpha *Expected\;value\;of\;lottery=\alpha *y_{t}/2 \end{array}$$

If α = 1, the individual is risk neutral. Another heuristic which is observationally equivalent is the rule that **Certainty equivalent** = θ ^*^ *y*_*t*_, where θ = α/2. Our setting is conducive to observing the proportional discounting heuristic, because of the price list format and the sequence of presentation of the choices. This is because if a subject switches from the safe choice *x*_*jt*_ to the risky choice *y*_*t*_ at the same row on the table in all periods, his behavior is consistent with the heuristic. Thus, an individual who wishes to apply the heuristic would not find it excessively cognitively demanding to do so. The average α parameter for this subsample is 0.92, equalling 0.96 for male and 0.90 for female subjects.

It is possible, if individuals have reference-dependent preferences, that α can differ between the domains of losses and gains, as proposed by Iturbe-Ormaetxe et al. (2011), Iturbe-Ormaetxe Kortajarene et al. (2015). Such a shift in the discount proportion can be seen in the right panel of Figure 2. This behavior reveals a discrete change in attitude toward risk above vs. below the reference point. However, in data such as ours, a classification of individuals according to the behavioral rules they employ, such as the Proportional Discounting Heuristic, must allow for some trials to exhibit deviations from the exact decision consistent with the heuristic. To classify individuals as users of the Proportional Discounting Heuristic, we calculate the following:

(2) $$\begin{array}{l} \Delta \;proportional\;valuation=(certainty\;equivalent/expected\;value\\ \;lottery{)}_{t}-(certainty\;equivalent/\\ \;expected\;value\;lottery{)}_{t\;-\;1}\\ \;x_{jt}^{*}/(0.5*y_{t})-x_{j,t\;-\;1}^{*}/(0.5*y_{t\;-\;1}),x_{jt}^{*}\\ \;=min_{j}\{x_{jt}|x_{jt}≽0.5*y_{t}\} \end{array}$$

**Figure 4:**
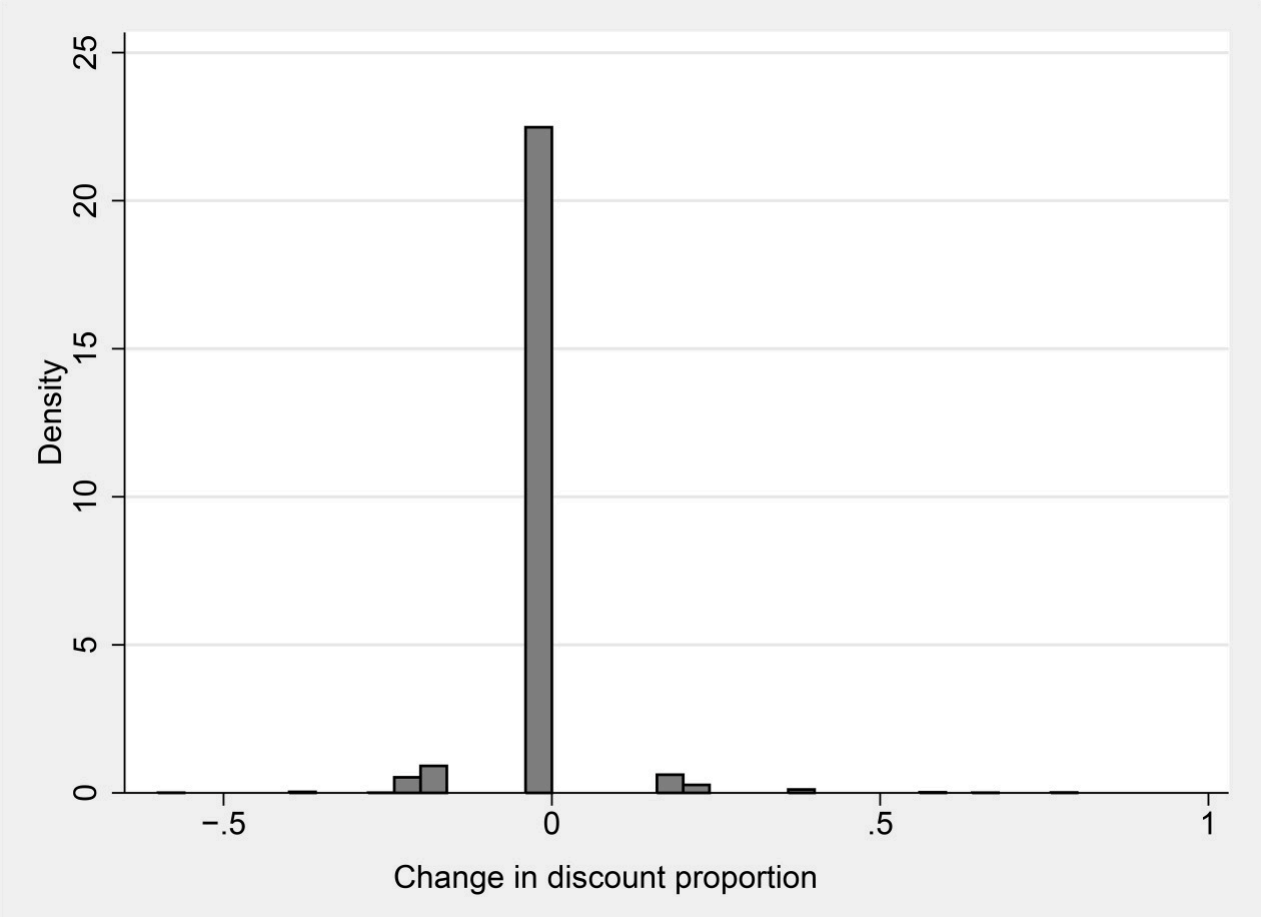
**Density of changes in discount proportion parameter α between periods ***t*** and ***t***+1**.

If the agent uses the proportional valuation heuristic, valuing every lottery at the same constant fraction of its expected value, then Δ *proportional valuation* always equals zero. We classify an individual as a proportional discounter if she exhibits no more than six instances over the 60-period session, in which Equation (2) does not equal 0. Figure 4 illustrates the stability of the strategy employed on the part of users of the heuristic. The figure is a histogram of (Δ *proportional valuation*) for the 38% of the sample that are proportional discounters. The change in proportional valuation is zero in the great majority of cases.

### 3.2. Reference points employed

To identify the reference points subjects are using, we focus on the manner whereby a reference point influences decisions. We test for the presence of a target payoff level by investigating the choice between playing the lottery and receiving the certain payment. We expect that the presence of a reference point will influence decisions when the certain payment is just above the reference level. In such cases, agents might forego some expected payoff and choose the certain payment, in order to reach their reference payoff. To test for this pattern, we model the choice between the certainty equivalent and the lottery of each individual as a function of the value of the certainty equivalent, the expected value of the lottery and a dummy variable indicating whether the safe option *x*_*jt*_ exceeds the reference point.

(3) $$\begin{array}{l} Z_{ijt}=\alpha_{i}+\beta_{1,i}0.5*y_{t}+\beta_{2,i}x_{jt}+\gamma_{k}D_{k}+\epsilon \end{array}$$

where

$$D_{k}=\{\begin{array}{l} 1;if\;Certain\;amount\;>\;reference\;point\;k\\ 0;if\;Certain\;amount\;\leq \;reference\;point\;k \end{array}$$

*Z*_*ijt*_ is a binary variable which represents the choice of individual *i* between the prospect (0.5, *y*_*t*_), and the certain amount on offer, *x*_*jt*_, in period *t*. *Z*_*ijt*_ takes the value 1 if the individual chooses the prospect, and 0 otherwise. Recall that all reference points are net of the initial endowment. A significant coefficient for the γ_*k*_ term would indicate the use of reference point *k*, as it reveals a change in the likelihood of choosing the lottery when the certain payment it is paired with exceeds the reference level. In the regression, we control for the expected value of the lottery and the level of the certain payment.

The model is estimated for each individual *i* and each reference point *k* separately. An *F*-test is performed to test for the significance of the restriction *D*_*k*_ = 0. If the resulting F-statistic is above the critical level, and the estimated gamma coefficient is negative, we will say that *k* is a reference point for the individual. When this test is significant for candidate reference point *k*, we say that the individual is using *k* as a reference point. Based on the result of this test, we assign an individual to either none, one, or multiple reference points. For each individual, the regression is estimated for each of the potential reference points. Table 2 shows the incidence of each possible reference point profile in the sample.

**Table 2:** **Reference point use by subjects**.

	**Session**	**All sample (%)**	**Female (%)**	Male^*^ (%)
	2–24			
None		17.83	16.66	20.57
Population Average (PA)		15.05	23.29	10.26
Individual Expectation (IE)		21.93	26.69	20.52
Historical Average (HA)		8.23	6.69	7.69
PA and IE		2.75	3.34	2.58
PA and HA		34.21	23.34	38.39
IE and HA		0	0	0
All		0	0	0
	25–44			
None		26.61	37.42	17.79
Population Average (PA)		62.27	52.53	73.38
Individual Expectation (IE)		2.23	0	2.23
Peer Expectation (PE)		0	0	0
PA and IE		8.88	10.05	6.61
PA and PE		0	0	0
IE and PE		0	0	0
All		0	0	0

* *The gender variable contains 5 missing values*.

The table shows that the PA is the most common reference point for individuals who used only one reference level, followed by IE and HA. PE does not seem to serve as a reference point. A sizable portion of subjects use multiple reference points, and most of these individuals use PA paired with HA. Lastly, a non-negligible portion of individuals do not appear to employ any of the candidate reference points. Gender differences are not significant, with Fisher exacts tests resulting in *p*-values of 0.61 for sessions 2–24, and 0.097 for sessions 25–44.

Regressions with the specification in Equation (3) on the aggregate pooled data from all individuals classified as using each reference point provide an overall picture of the estimated parameters, and of the strength of the attraction of each reference point. Recall that each reference point, other than PA, is specified as in addition to the initial endowment. The estimates are shown in Tables 3, 4. The results show that an increase in the expected value of the lottery increases the probability of choosing the lottery. On the other hand, increasing the value of the certain alternative decreases the probability of choosing the lottery. Each of the reference points is negative and significant in both tables. This indicates that for each of the reference points PA, HA, and IE, a subset of subjects exhibits changes in behavior for payoff levels above vs. below the reference point. When the certain payoff exceeds the reference point, it is more likely to be chosen.

**Table 3:** **Estimated effect of reference point in sessions 2–24**.

	**(1) choice**	**(2) choice**	**(3) choice**
EV Lottery (0.5*y_*t*_)	0.05^***^	0.09^***^	0.06^***^
	(7.48)	(14.04)	(10.39)
*x_jt_*	−0.05^***^	−0.07^***^	−0.06^***^
	(−9.55)	(−16.09)	(−13.61)
*D_PA_*	−0.42^***^		
	(−16.01)		
*D_IE_*		−0.37^***^	
		(−15.69)	
*D_HA_*			−0.35^***^
			(−11.04)
Gender	−0.05	−0.02	−0.03
	(−1.51)	(−0.54)	(−0.84)
Constant	0.61^***^	0.49^***^	0.64^***^
	(19.95)	(13.65)	(18.02)
Observations	16,720	8616	12,896
(*R*^2^)	0.514	0.544	0.538

*t statistics in parentheses*.

*Robust standard errors*.

*** *(p < 0.01)*.

**Table 4:** **Estimated effect of reference point in sessions 25–44**.

	**(1) choice**	**(2) choice**
EV Lottery (0.5*y_*t*_)	0.06^***^	0.07^***^
	(11.63)	(11.00)
*x_jt_*	−0.05^***^	−0.05^***^
	(−13.85)	(−14.01)
*D_PA_*	−0.46^***^	
	(−26.64)	
*D_IE_*		−0.33^***^
		(−16.01)
Gender	0.02	−0.01
	(0.81)	(−0.32)
Constant	0.56^***^	0.43^***^
	(22.18)	(9.91)
Observations	29,192	3824
(*R*^2^)	0.552	0.579

*t statistics in parentheses*.

*Robust standard errors*.

*** *(p < 0.01)*.

### 3.3. Income shock

In the Shift treatment, we study the effect of a shock to an individual's income level and investigate whether it changes the likelihood of choosing a particular reference point. In this treatment, at the end of period 40, subjects experience a change in their wealth. We increase their cash balance by 50% of their initial endowment, an amount which differs among subjects. Then, in the last 20 periods of the session, the same set of choices as in the first 20 periods are presented to the subjects again. We consider the effect of the shock on the choices of individuals in the last 20 periods of the experiment and compare these to the choices elicited in the first segment of 20 periods, with respect to which reference points most accurately characterize the decision pattern.

We report the proportions of reference points that fit best the decisions of these individuals in Table 5. The first column reports a classification of individuals in relation to reference points in periods 1–20 in the Shift treatment. The second column contains analogous data from periods 41–60. The results show no significant difference in the incidence of the use of each reference point before, compared to after, the shock. A Fisher exact test of the equality of the distribution of reference points between periods 1–20 and 41–60 results in *p* = 0.481. This may reflect the fact that the shock, like initial income, is treated as a separate source of wealth than the earnings from the experimental task.

**Table 5:** **Reference points of subjects in Shift treatment before and after the income shock**.

	**Period 1–20 (%)**	**Period 41–60 (%)**
None	36.07	41.00
Population Average (PA)	59.00	57.35
Individual Expectation (IE)	1.64	0
Peer Expectation (PE)	3.29	0
PA and IE	0	0
PA and PE	0	0
IE and PE	0	1.64
All	0	0

## 4. Discussion

In this paper, we document heterogeneity among individuals in their personal inclination to use particular reference points. It is known from previous work that the reference point that characterizes a set of data best differs, depending on the setting in which the decision is taking place. However, we show here that the reference point that best fits the decision pattern of an individual also differs by individual, keeping the decision setting constant.

Our results do indicate that when individuals use a single reference point, the population average payoff level is the most frequently employed. This is followed by the anticipated payoff level indicated for the individual, and in turn by the average that comparable individuals have earned in past similar tasks. No participant used the earnings of peers in the same session as a reference point. The results are similar for men and women and we observe no significant gender differences in the use of reference points.

We also observe that a sizable fraction of individuals employs multiple reference points. The most common combinations of reference points are the population average with the historical average, and the population average with the individual expectation. It is striking to us that PA is such a strong attractor, in light of the fact that the social distance between an individual and the population average is arguably the greatest among all of the reference points that we have considered. The experimental design we have does not allow us to isolate the precise reason that PA is more prominent than the others. However, it does have the feature that it, along with HA, is historical and therefore certain, while IE and PE are anticipated future payoff levels. Furthermore, PA is always constant and known to be the same for all individuals, while the three other reference points can vary among individuals. Perhaps a reference payoff is more compelling when it is common knowledge that it is the same for everyone.

We also find that a considerable share of subjects tend to proportionally discount their certainty equivalent by a constant percentage of the expected payoff of the risky lottery. Some of these individuals also discount by a different fraction, depending on whether payoffs are above or below one or more of the reference points. The widespread use of the Proportional Discounting Heuristic seems intuitive as a behavioral rule, because it is simple to calculate and apply, though to our knowledge its use has not been documented in previous research.

Thus, our experiment illustrates two types of heterogeneity in how individuals perceive risky decision making tasks. The first is that some individuals differ in whether or not they apply a simple heuristic, proportional discounting, to value the lottery, while others adopt more complex or inconsistent valuation methods. The second is that the reference level of earnings that individuals use is idiosyncratic, with some individuals targeting one or more from among a set of prominent reference points, while others do not.

While a number of studies have focused on estimating the mean and median loss aversion parameters of a particular sample, a growing number of studies have documented heterogeneity in the loss aversion level of individuals (Fehr and Goette, 2007; Gächter et al., 2007; Von Gaudecker et al., 2011). Building on this, other studies have investigated factors affecting the degree of individual loss aversion and have found that demographic characteristics play an important role (Hjorth and Fosgerau, 2009; Payne et al., 2015). Loss aversion only has meaning relative to a reference point. Our results complement this line of research by providing evidence that individuals exhibit different reference points in a similar task. Thus, in addition to having different levels of loss aversion, the reference points from which loss aversion is defined, are heterogeneous.

## Author contributions

AT conducted the experiment and analyzed the data. All four authors contributed to designing the experiment, guiding the analysis of the results, and writing the paper.

## Funding

The Center of Economic Research at Tilburg University provided the funds used to compensate participants in our experiment.

### Conflict of interest statement

The authors declare that the research was conducted in the absence of any commercial or financial relationships that could be construed as a potential conflict of interest.

## Footnotes

^1^Paying only one period removes wealth effects. Starmer and Sugden (1991) have shown that this procedure generates behavior that is similar to that when all periods are paid.

^2^One measure of consistency of choices that can be applied to the data is whether subjects' certainty equivalent increases from one period to the next within each 20-period segment. By this criterion, 14 subjects are consistent for all 60 decisions, 46 have fewer than 5 inconsistencies, and 98 have fewer than 10.
